# Comparisons between multi-component myelin water fraction, T1w/T2w ratio, and diffusion tensor imaging measures in healthy human brain structures

**DOI:** 10.1038/s41598-019-39199-x

**Published:** 2019-02-21

**Authors:** Md. Nasir Uddin, Teresa D. Figley, Kevin G. Solar, Anwar S. Shatil, Chase R. Figley

**Affiliations:** 1 Department of Radiology, University of Manitoba, Winnipeg, MB Canada; 2 Division of Diagnostic Imaging, Health Sciences Centre, Winnipeg, MB Canada; 3 Neuroscience Research Program, Kleysen Institute for Advanced Medicine, Winnipeg, MB Canada; 4 Department of Physiology and Pathophysiology, University of Manitoba, Winnipeg, MB Canada; 5 Biomedical Engineering Graduate Program, University of Manitoba, Winnipeg, MB Canada; 6 Department of Psychological and Brain Sciences, Johns Hopkins University, Baltimore, MD USA

## Abstract

Various MRI techniques, including myelin water imaging, T1w/T2w ratio mapping and diffusion-based imaging can be used to characterize tissue microstructure. However, surprisingly few studies have examined the degree to which these MRI measures are related within and between various brain regions. Therefore, whole-brain MRI scans were acquired from 31 neurologically-healthy participants to empirically measure and compare myelin water fraction (MWF), T1w/T2w ratio, fractional anisotropy (FA), axial diffusivity (AD), radial diffusivity (RD) and mean diffusivity (MD) in 25 bilateral (10 grey matter; 15 white matter) regions-of-interest (ROIs). Except for RD vs. T1w/T2w, MD vs. T1w/T2w, moderately significant to highly significant correlations (p < 0.001) were found between each of the other measures across all 25 brain structures [T1w/T2w vs. MWF (Pearson r = 0.33, Spearman ρ = 0.31), FA vs. MWF (r = 0.73, ρ = 0.75), FA vs. T1w/T2w (r = 0.25, ρ = 0.22), MD vs. AD (r = 0.57, ρ = 0.58), MD vs. RD (r = 0.64, ρ = 0.61), AD vs. MWF (r = 0.43, ρ = 0.36), RD vs. MWF (r = −0.49, ρ = −0.62), MD vs. MWF (r = −0.22, ρ = −0.29), RD vs. FA (r = −0.62, ρ = −0.75) and MD vs. FA (r = −0.22, ρ = −0.18)]. However, while all six MRI measures were correlated with each other across all structures, there were large intra-ROI and inter-ROI differences (i.e., with no one measure consistently producing the highest or lowest values). This suggests that each quantitative MRI measure provides unique, and potentially complimentary, information about underlying brain tissues – with each metric offering unique sensitivity/specificity tradeoffs to different microstructural properties (e.g., myelin content, tissue density, etc.).

## Introduction

The microstructural integrity of brain tissue is thought to play a crucial role in healthy brain function, and is also presumed to have a direct (causal) relation to clinical symptoms among patients with brain injury or neurological disease^1,2^. In particular, the integrity of myelinated axons – and white matter (WM) in general – is essential for efficient communication between different brain regions and facilitating normal brain function. However, neurodegenerative diseases such as multiple sclerosis (MS), neuromyelitis optica, and schizophrenia can lead to demyelination and other microstructural tissue changes^3–6^. Therefore, due to its three-dimensional nature and ability to endogenously (non-invasively) sensitize signals to different characteristics and various pathologies – e.g., using myelin water imaging (MWI), diffusion tensor imaging (DTI), and other quantitative methods^7–17^ – magnetic resonance imaging (MRI) has become the most common approach for characterizing brain tissue microstructure *in vivo*.

Although alternative MWI approaches exist – e.g., multi-component driven equilibrium single pulse observation of T1 and T2 (mcDESPOPT)^18^ – obtaining myelin water fraction (MWF) estimates from a 3D gradient echo spin echo (GRASE) sequence has become one of the most validated and common quantitative measures for non-invasively measuring myelin content in the brain^12^. This method is based on spin-spin relaxation curves obtained from a series of T2-weighted (T2w) images with multiple echo times. Typically, three T2 components are then modeled in the T2 distribution, including short, intermediate and long components that are attributed to water trapped between myelin lipid bilayers (T2 < 40 ms), intra/extracellular water (T2 ≈ 40–200 ms), and cerebrospinal fluid (T2 > 2000 ms), respectively. The MWF is then calculated as the ratio of the short T2 relaxation component (e.g., between 10–40 ms) relative to the total T2 distribution^12,19–21^. Previous studies have shown that the MWFs derived in this way correlate strongly with ‘gold standard’ histological measures of myelin concentration^22–24^, and that the resulting MWFs are also relatively insensitive to inflammation and other non-myelin-related aspects of pathology in diseased tissues^25^. However, the major limitations of MWI are the long scan times required for whole-brain acquisition, the relatively complex (and computationally intensive) post-processing, and inherently low contrast-to-noise ratios (CNR) of the MWF maps – especially in regions with relatively low myelin content^12^.

In part to circumvent these limitations, it was recently proposed that dividing T1-weighted images by T2-weighted images (i.e., to generate a T1w/T2w ratio map) could be used to assess tissue microstructure (specifically myelin concentration) in a relatively straightforward manner. This method was initially proposed for mapping intra-cortical myelin^26^, but several calibrated approaches have since been developed to facilitate whole brain applications^27–29^. The underlying premise is that myelin and inflammation alter the signal intensity of T1w and T2w images in opposite directions, so T1w/T2w ratio maps should provide both increased tissue contrast and better sensitivity related to myelin content and tissue microstructure in brain tissue. Moreover, although raw T1w and T2w images (and raw T1w/T2w ratios) are not inherently quantitative since their intensities vary between scanners and sessions, they can be bias-corrected and intensity-scaled (relative to reference structures outside of the brain) in order to generate quantitative maps with high reproducibility and low inter-subject variability^29^. To date, the T1w/T2w method has been used to assess myelin content within the grey matter (GM) of the cerebral cortex^26,30–32^, estimate myelin contents in WM of neonatal brains^33^, quantify MS-related tissue damage within normal appearing white matter (NAWM)^27^, assess abnormalities in schizophrenia^34,35^ and bipolar disorders^36^, and even as a biomarker for amyloid beta accumulation^37^. The major advantage of this method is the use of common T1w and T2w images that are already acquired in practically all clinical and research brain MRI protocols, and the T1w/T2w method has some inherent theoretical advantages over diffusion-based metrics as well. For example, T1w/T2w signal intensities are not affected by fiber orientation and might therefore provide more sensitive and reliable measures compared to diffusion-based metrics – particularly in regions with crossing-fibers and other complex fiber configurations. Moreover, the same data (i.e., T1w images) can be used to perform complimentary volumetric and cortical thickness measurements^26,28^, while another recent study has shown that T2w images from a MWI-based GRASE sequence can be used (instead of T2w fast spin echo images) to calculate T1w/T2w ratios from the same T2w data^38^. Therefore, T1w/T2w ratios have several attractive qualities, and even though they might not be particularly specific to myelin concentrations in WM regions, recent studies by our lab and others have demonstrated that T1w/T2w measurements might still be useful as general measures of tissue microstructure^38–40^.

On the other hand, DTI provides quantitative measures to characterize tissue microstructure based on the application of multiple diffusion gradients to probe molecular water diffusion in the brain – typically in a large number of different directions^41–44^. DTI metrics such as fractional anisotropy (FA), axial diffusivity (AD), radial diffusivity (RD) and mean diffusivity (MD) are considered to be indicators of WM microstructure owing to their sensitivities to cellular density, axonal size, water content, myelin content and other tissue properties^41,45^. In particular, FA reflects the degree of non-isotropic diffusion (which can be used to infer the degree to which water-restricting barriers are aligned, as well as the principal direction of that alignment); and although FA is extremely sensitive to microstructural changes, it is not thought to be specific to any particular tissue characteristics and/or pathologies^46^. Similarly, AD tends to be variable between brain regions and across a range of WM pathologies, whereas RD is generally thought to increase with de-myelination and/or reduced axon density^46^. MD, which is the weighted mean of AD and RD, is a measure of the average magnitude of water diffusion (which can be used to infer the overall density of tissue barriers) within a given voxel or brain region^43,44,47^. However, because different types of tissue boundaries and several general factors (e.g., temperature, inflammation, etc.) influence diffusion-based measurements, these are thought to be relatively broad (as opposed to myelin-specific) indicators of tissue microstructure. Moreover, due to the inherently directional nature of FA, AD and RD measurements, these values are highly prone to artefacts resulting from partial volume averaging between different WM tracts (i.e., with crossing fibers or other complex geometries).

In order to establish the extent to which these methods reflect similar tissue characteristics, and to determine whether some methods may be more appropriate than others for investigating specific pathologies, several previous studies have been done to compare different quantitative MRI measures in human brains^29,38–40,48–50^. For example, Madler *et al*. performed a quantitative comparison of MWF and DTI metrics (i.e., FA, MD and apparent diffusion coefficient ADC) in 11 WM and GM structures, finding strong correlations between the measures across all structures (r = 0.87 for MWF vs. FA, r = 0.74 for MWF vs. ADC)^48^. Ganzetti *et al*. compared the reproducibility of calibrated T1w/T2w ratio values with magnetization transfer ratio (MTR), FA and fluid attenuated inversion recovery (FLAIR) intensities in 6 WM and 3 subcortical GM structures, finding that calibrated T1w/T2w ratios had high reproducibility (especially in WM structures)^29^. Arshad *et al*. have reported moderate correlations between MWF and T1w/T2w (r = 0.21 to r = 0.65) within various WM structures, but a negative overall correlation after combining data across structures (r = −0.26)^39^. Recent studies by our lab also compared the relationship between MWF and T1w/T2w ratios in subcortical structures. Using a cohort of MS patients with a wide age range (57 ± 18 years), we found extremely low correlations across WM structures (r = 0.004), low correlations across all structures (r = 0.23), and moderate correlations among subcortical GM structures (r = 0.45)^38,40^, and similar correlations were found for relatively young (age 29 ± 11 years), neurologically-healthy participants as well^40^. Using a cohort of healthy children, Geeraert and coworkers recently compared quantitative inhomogeneous magnetization transfer (qiMT), myelin volume fraction (MVF) using mcDESPOT, and RD using DTI, finding strong correlations between measures (r = 0.89 for qihMT vs. MVF, r = −0.66 for RD vs. MVF, r = −0.74 for RD vs. MVF)^49^. Finally, a very recent study by Ercan *et al*. compared inhomogeneous magnetization transfer ratio (ihMTR), T2-relaxation based MWF, and DTI metrics (FA, AD, RD, and MD), reporting a wide range of correlations between various measures (r = 0.77 for ihMTR vs. MWF, r = −0.30 for ihMTR vs. RD, r = 0.20 for ihMTR vs. FA, r = −0.19 for ihMTR vs. MD, r = 0.02 for ihMTR vs. AD)^50^. However, to the best of our knowledge, no studies to date have directly compared calibrated T1w/T2w ratios with both T2-relaxation-based MWFs (i.e., myelin-specific measures) and diffusion-based FA, AD, RD and MD (i.e., general measures of tissue microstructures) in the same cohort – which is important for validating/replicating recent studies showing that T1w/T2w ratios may not be myelin-specific (particularly in subcortical WM structures), and for testing the notion that T1w/T2w signals might be similar to diffusion-based MRI metrics.

In this work, we therefore used six different MRI measures, including MWF, T1w/T2w, FA, AD, RD and MD to analyze brain tissue properties – both to compare them to each other and to establish normative values within a number of different brain structures. The main goals of the current work were to: (1) verify previous work comparing T1w/T2w ratio vs. MWF in a larger sample population and in more brain structures^38–40^, and (2) directly compare T1w/T2w ratios to various DTI metrics. In addition to potentially shedding light on the mechanisms underlying T1w/T2w ratio measurements in subcortical structures, this could also have practical implications for future multi-modal imaging studies – both in terms of prospective study planning (e.g., by informing the choices of pulse sequences to be included in study protocols) and in terms of post-hoc data analysis (e.g., by reducing the number of redundant multiple comparisons in multi-modal neuroimaging studies that can potentially arise by analyzing data using different quantitative imaging metrics).

## Methods

### Participants

Thirty-one healthy volunteers (15 males, 16 females) aged 18–57 years (29.6 ± 10.7 years) were enrolled from the Charles Village and Roland Park communities in Baltimore, Maryland, USA. Written informed consent was obtained from each volunteer, and all experiments were performed in accordance with the relevant guidelines and regulations of the Johns Hopkins Medical Institutions and Johns Hopkins University institutional review boards. All participants were verbally screened to confirm the absence of any current or previous neurological disorder or psychiatric disease. Participant ages (males 28.3 ± 9.9 years; females 30.9 ± 11.5 years) were not significantly different between the two groups (p = 0.47).

### Data Acquisition

Participants were scanned using a whole-body 3 T Philips Achieva MRI system equipped with a 32-channel SENSE head coil (*Philips Healthcare, Best, The Netherlands)*.

#### T1-weighted imaging

High-resolution T1-weighted images were acquired using a three-dimensional (3D) Turbo Field Echo (TFE) pulse sequence with the following parameters: Repetition Time [TR] = 7.93 ms; Echo Time [TE] = 3.66 ms; Flip Angle = 8°; SENSE Factor = 2.4; Field Of View [FOV] = 212 mm × 150 mm × 172 mm; Spatial Resolution = 1.00 mm × 1.00 mm × 1.00 mm; Scan Duration = 4:26 min.

#### Myelin water imaging

MWI scans used a whole-cerebrum 32-echo 3D gradient and spin echo (GRASE) sequence^12^ with the following parameters: TR = 1500 ms; Echo Train Lengths [ETL] = 32; Echo Spacing [ESP] = 10.36 ms; first Echo Times [TE1] = 10 ms; Number of Slices = 32; Slice Thickness = 3 mm; EPI Factor = 3 (in the z-direction); SENSE Factor = 4.0; FOV = 212 mm × 212 mm × 96 mm; Spatial Resolution = 0.95 mm × 0.95 mm × 3.00 mm; Scan Duration = 7:29 min.

#### Diffusion tensor imaging

DTI was performed using a single-shot spin echo, echo-planar imaging (SE-EPI) pulse sequence with the following parameters: 30 diffusion-encoded images (b = 700 s/mm^2^); 5 reference images (b = 0 s/mm^2^); TR = 6904 ms; TE = 69 ms; Flip Angle = 90°; SENSE Factor = 2.5; FOV = 212 mm × 154 mm × 212 mm; Number of Transverse Slices = 70 (no inter-slice gap); Spatial Resolution (Acquired) = 2.20 mm × 2.20 mm × 2.20 mm; Spatial Resolution (Resampled) = 0.83 mm × 0.83 mm × 2.20 mm; Scan Duration = 4:16 min.

### Image Processing

All images were analyzed using the SPM12 *(*http://www.fil.ion.ucl.ac.uk/spm/software/spm12/*, Wellcome Trust Centre for Neuroimaging, London, UK), MRIStudio (*https://www.mristudio.org/*, Johns Hopkins University School of Medicine, Baltimore, Maryland, USA)* and custom in-house MATLAB R2017a (The Mathworks Inc., *Natick, MA, USA*) programs.

For each participant, all of the GRASE data (T2w images acquired with 32 different TEs) were coregistered to the T1w TFE images and then resliced to 1 mm^3^ resolution using “resize_img.m” ﻿script (http://www.cs.ucl.ac.uk/staff/G.Ridgway/vbm/resize_img.m*, University College London, UK*). Then, skull stripping of all the images was performed by generating participant-specific brain masks in SPM12, and refining the masks manually using the ROIEditor Toolbox in MRIStudio. The coregistered and skull-stripped mean images were then normalized to the *“JHU_MNI_SS_T1_ss”* template^51^ in Montreal Neurological Institute (MNI) coordinate space^52^ using a 12-parameter affine (linear) transformation with Automated Image Registration (AIR), followed by high-dimensional, non-linear warping with the large deformation diffeomorphic metric mapping (LDDMM) algorithm with alpha values of 0.01, 0.005, and 0.002^53^ in MRIStudio’s DiffeoMap Toolbox, as previously reported^54^. The alpha values constrain the amount of elasticity allowed in each iteration of the deformation, so using three iterations with cascading alpha values allows for increasingly non-linear registrations. The T1w and GRASE images were then spatially normalized to MNI space by applying the linear (affine) and non-linear LDDMM transformations before generating voxel-wise MWF maps using a regularized non-negative least squares approach and an extended phase graph algorithm to compensate for any stimulated echoes due to B1 heterogeneities^12,55^. In this way, MWF was calculated based on the ratio of T2w signal between 10–40 ms to the total T2w distribution^19^.

Whole-brain calibrated T1w/T2w maps were generated for each participant using the T1w TFE image and the T2w GRASE image with TE = 140 ms, as recently recommended for GRASE-based T1w/T2w calculations^40^. Bias correction and calibration were performed to all of the images using previously described methods^29^, after co-registering T2w GRASE images with T1w TFE images. Intensity calibration was performed in native-space and without skull-stripping because the procedure requires signals from eyeballs and temporal muscles, but binary masks – obtained using T1w images and FSL’s brain extraction tool (BET)^56^ with a fractional intensity threshold of 0.45 – were then applied to the resulting T1w/T2w ratio maps. The skull-stripped T1w/T2w maps were then spatially normalized to MNI space by applying the same linear (affine) and LDDMM-based deformation described above.

After coregistering each participant’s diffusion weighted data with T1w anatomical images, DTI images were preprocessed using CATNAP (Coregistration, Adjustment, and Tensor-solving, a Nicely Automated Program; http://iacl.ece.jhu.edu/~bennett/catnap/, *JHU School of Medicine, Baltimore*, *Maryland, USA*) to correct for motion artifacts and coregister the DTI images to the reference images (i.e., the mean b = 0 s/mm^2^ image) using 12-parameter (affine) registration, which additionally corrects for eddy current distortions^13^. CATNAP then automatically calculated the six tensor images (dxx, dyy, dzz, dxy, dxz, dyz) and three diagonalized eigenvalue images (λ_1_, λ_2_, λ_3_) using a log-linear minimum mean squared error (LLMMSE) approach^57^, which assumes that noise is independently and identically distributed in a Gaussian fashion (as previously supported for SNR values greater than 2:1)^58^. After skull-stripping the mean b = 0 s/mm^2^ image and six tensor images, the same linear (affine) and non-linear LDDMM-based approach (described above) was applied to spatially normalize the mean b = 0 s/mm^2^ image to the *“JHU_MNI_SS_b0_ss”* template in MNI space, and the resulting deformation field was applied to spatially normalize the three eigenvalue images before finally generating voxel-wise fractional anisotropy (FA), axial diffusivity (AD), radial diffusivity (RD), and mean diffusivity (MD) maps using the DTIStudio Toolbox^59^ in MRI studio.

#### Region of interest segmentation

**Figure 1:**
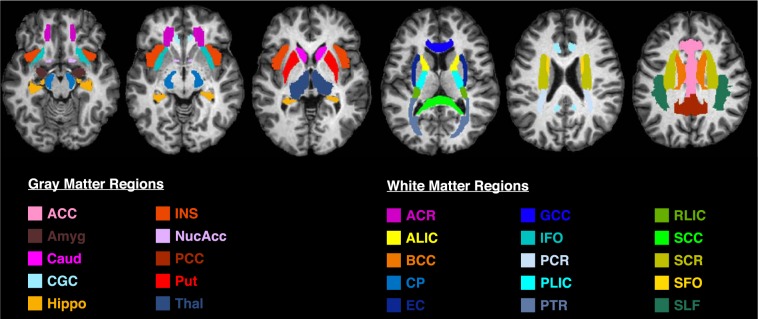
T1-weighted axial MPRAGE image with 25 regions of interest obtained using the JHU_MNI_SS (‘Eve’) atlas. Colors indicate different brain structures. The 15 white matter structures include - ACR: Anterior Corona Radiata, SCR: Superior Corona Radiata, CP: Cerebral Peduncle, PLIC: Posterior Limb of Internal Capsule, ALIC: Anterior Limb of Internal Capsule, RLIC: Retrolenticular part of Internal Capsule, PTR: Posterior Thalamic Radiation, PCR: Posterior Corona Radiata, IFO: Inferior Fronto-Occipital Fasciculus, SFO: Superior Fronto-Occipital Fasciculus, SLF: Superior Longitudinal Fasciculus, BCC: Body of Corpus Callosum, EC: External Capsule, GCC: Genu of Corpus Callosum, SCC: Splenium of Corpus Callosum. The 10 grey matter structures include - ACC: Anterior Cingulate Cortex, PCC: Posterior Cingulate Cortex, Caud: Caudate nucleus, CGC: Cingulate Gyrus (Cortex), INS: Insular cortex, Thal: Thalamus, Put: Putamen, Hippo: Hippocampus, Amyg: Amygdala, and NuAcc: Nucleus Accumbens.

After generating the MWF, T1w/T2w, FA, AD, RD and MD maps, region-of-interest (ROI) analyses were performed for each participant using MRIStudio’s ROIEditor Toolbox. 3D ROIs, listed in the JHU_MNI_SS (‘Eve’) atlas, were chosen for 25 brain regions, including 15 WM structures and 10 subcortical grey matter (GM) structures (Fig. 1). The investigated WM structures included the ACR: Anterior Corona Radiata, SCR: Superior Corona Radiata, CP: Cerebral Peduncle, PLIC: Posterior Limb of Internal Capsule, ALIC: Anterior Limb of Internal Capsule, RLIC: Retrolenticular part of Internal Capsule, PTR: Posterior Thalamic Radiation, PCR: Posterior Corona Radiata, IFO: Inferior Fronto-Occipital Fasciculus, SFO: Superior Fronto-Occipital Fasciculus, SLF: Superior Longitudinal Fasciculus, BCC: Body of Corpus Callosum, EC: External Capsule, GCC: Genu of Corpus Callosum, SCC: Splenium of Corpus Callosum. The investigated GM structures included the ACC: Anterior Cingulate Cortex, PCC: Posterior Cingulate Cortex, Caud: Caudate nucleus, CGC: Cingulate Gyrus (Cortex), INS: Insular cortex, Thal: Thalamus, Put: Putamen, Hippo: Hippocampus, Amyg: Amygdala, and NuAcc: Nucleus Accumbens.

For bilateral structures, MWF, T1w/T2w ratio, FA, AD, RD and MD values were averaged over both the left and right hemispheres to yield a mean value for each metric, ROI, and participant (i.e., 6 metrics × 25 ROIs × 31 participants = 4,650 unique data points).

#### Statistical analysis

After extracting all of the raw values from each brain region, three types of statistical analyses were performed, and all statistical analyses were performed using MATLAB 2017a (*The Mathworks Inc., Natick, MA, USA*) and SPSS version 20 (*IBM Inc., Armonk, NY, USA*).

First, paired-sample t-tests were performed to investigate overall differences between tissue types (i.e., WM vs. subcortical GM) for each MRI metric. In order to achieve this, raw values for each metric were averaged across all 15 WM structures (i.e., to generate a mean WM value) and all 10 subcortical GM structures (i.e., to generate a mean GM value) for each participant. Paired t-tests were then performed using the corresponding WM and GM values from each participant; and due to strong *a priori* directional hypotheses (e.g., MWF higher in WM than GM, etc.), one-tailed t-tests were used. Given that there are 6 different MRI metrics, the overall type-I error rate (due to multiple comparisons) for each of the paired-sample, one-tailed t-tests was controlled by applying a post-hoc family-wise error (Bonferroni) correction – i.e., requiring a threshold of p < 0.008 (0.05/6) for any given t-test to be deemed statistically significant.

Second, Pearson (linear) and Spearman (rank) correlations were initially used to examine the relationships between the different MRI measures after combining data points across all 25 structures, across only the 15 WM structures, and across the 10 subcortical GM structures – in order to reveal overall trends (i.e., including potentially large differences in tissue properties between structures). However, Pearson and Spearman correlations were then performed to investigate the relationships between different MRI measures within each structure separately. Given that there are 15 unique between-method comparisons, the overall type-I error rate (due to multiple comparisons) for each of the Pearson and Spearman correlations was controlled by applying a post-hoc family-wise error (Bonferroni) correction – i.e., requiring a threshold of p < 0.003 (0.05/15) for any given Pearson or Spearman correlation to be deemed statistically significant.

Finally, in order to facilitate comparisons between measures with different intensity scales and/or units, the raw values of each MRI measure (i.e., MWF, T1w/T2w ratio, FA, AD, RD and MD) were standardized (z-scored) across all 25 structures. However, before calculating the z-scores, values for RD and MD were inverted (i.e., 1/RD and 1/MD, denoted as RD^−1^ and MD^−1^), in line with similar recent analyses^49^ in order to produce analogous contrasts (i.e., positive z-scores reflecting greater microstructural integrity and negative z-scores reflecting lower microstructural integrity) across measures. Repeated measures ANOVAs (aka, ANOVAs for correlated samples) were then performed independently for each brain region to test for differences (F-statistics) between the mean z-scores of the six quantitative metrics. Given that there are 25 ROIs, the overall type-I error rate (due to multiple comparisons) was controlled by applying a post-hoc family-wise error (Bonferroni) correction – i.e., requiring a threshold of p < 0.002 (0.05/25) for any given F-statistic to be deemed statistically significant.

## Results

### ROI analysis and normative values

**Figure 2:**
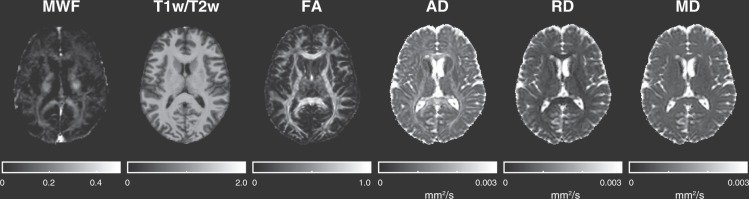
Example MWF, T1w/T2w ratio, and DTI metric (FA, AD, RD and MD) maps obtained from a representative healthy participant. Intensity scales are also shown below each image. Note MWF, T1w/T2w ratio, and FA are scalars while AD, RD and MD are in mm^2^/s.

Figure 1 shows a T1-weighted anatomical image with all 25 ROIs (15 WM structures and 10 subcortical GM structures) overlaid, Fig. 2 shows example MWF, T1w/T2w, FA, AD, RD and MD maps obtained from a representative healthy volunteer, and Table 1 lists the mean, standard deviation (SD), and coefficient of variation (COV; also known as relative standard deviation) between participants for the MWF, T1w/T2w ratio, FA, AD, RD and MD values in each ROI. Interestingly, the average COV across all structures was highest for MWF (COV = 19.5) and RD (COV = 19.4), followed by T1w/T2w ratio (COV = 14.9), FA (COV = 8.2), MD (COV = 4.8) and AD (COV = 4.7). Moreover, although measurement variability showed no apparent dependence on tissue type for T1w/T2w ratio, FA and MD measures (p > 0.05, uncorrected), COVs tended to be higher in subcortical GM structures for MWF and AD, and lower for RD (all three p < 0.02, uncorrected) compared to COVs from WM structures (based on two-tailed, two-sample t-tests).

**Table 1:** Mean, standard deviation (SD) and coefficient of variation (COV) MRI measures (MWF, T1w/T2w, FA, AD, RD, MD) for all 25 brain structures.

Structure	MWF (%)		T1w/T2w		FA		AD ×10^−3^ (mm^2^/s)		RD ×10^−3^ (mm^2^/s)		MD ×10^−3^ (mm^2^/s)	
	Mean ± SD	COV	Mean ± SD	COV	Mean ± SD	COV	Mean ± SD	COV	Mean ± SD	COV	Mean	COV
ACR	7.12 ± 1.35	18.95	0.90 ± 0.12	13.67	0.41 ± 0.03	7.30	1.23 ± 0.03	2.76	0.64 ± 0.13	20.26	0.82 ± 0.02	2.82
ALIC	11.42 ± 2.15	18.80	0.70 ± 0.09	12.34	0.49 ± 0.05	10.02	1.21 ± 0.05	4.28	0.53 ± 0.09	16.74	0.75 ± 0.02	3.12
BCC	9.61 ± 1.70	17.66	0.95 ± 0.13	13.32	0.59 ± 0.04	7.22	1.79 ± 0.11	6.00	0.62 ± 0.13	20.88	1.02 ± 0.07	6.64
CP	18.56 ± 1.92	10.33	0.99 ± 0.13	12.86	0.60 ± 0.03	5.38	1.77 ± 0.08	4.56	0.65 ± 0.14	20.99	1.02 ± 0.07	7.23
EC	5.30 ± 1.39	26.22	0.87 ± 0.11	12.54	0.38 ± 0.02	4.64	1.13 ± 0.02	2.19	0.64 ± 0.13	19.79	0.79 ± 0.02	2.48
GCC	9.59 ± 1.83	19.12	0.79 ± 0.11	14.04	0.65 ± 0.04	5.81	1.69 ± 0.08	4.50	0.51 ± 0.09	18.01	0.89 ± 0.03	3.82
IFO	3.88 ± 1.25	32.16	0.80 ± 0.12	15.50	0.44 ± 0.03	7.82	1.28 ± 0.04	3.00	0.62 ± 0.12	20.02	0.82 ± 0.02	2.42
PCR	10.27 ± 1.79	17.43	1.09 ± 0.15	13.49	0.42 ± 0.03	6.83	1.21 ± 0.05	4.20	0.64 ± 0.14	21.39	0.81 ± 0.03	3.49
PLIC	16.04 ± 1.36	8.49	1.05 ± 0.14	13.57	0.58 ± 0.03	4.51	1.32 ± 0.05	3.53	0.47 ± 0.09	18.14	0.75 ± 0.02	3.12
PTR	10.97 ± 1.37	12.51	0.90 ± 0.13	14.30	0.52 ± 0.3	4.90	1.47 ± 0.04	2.56	0.63 ± 0.14	21.57	0.89 ± 0.04	4.57
RLIC	10.95 ± 1.38	12.62	1.05 ± 0.16	15.47	0.52 ± 0.02	4.10	1.37 ± 0.04	2.86	0.57 ± 0.11	18.64	0.82 ± 0.02	2.68
SCC	11.81 ± 1.38	11.71	0.78 ± 0.12	14.99	0.61 ± 0.03	5.64	1.81 ± 0.08	4.62	0.64 ± 0.14	21.61	1.02 ± 0.07	6.77
SCR	11.16 ± 1.59	14.24	1.04 ± 0.13	12.89	0.42 ± 0.02	4.35	1.13 ± 0.03	2.76	0.59 ± 0.11	19.13	0.76 ± 0.02	2.89
SFO	8.81 ± 1.65	18.73	0.78 ± 0.14	17.76	0.28 ± 0.07	26.45	1.00 ± 0.08	8.27	0.65 ± 0.14	21.30	0.75 ± 0.05	7.05
SLF	10.75 ± 1.52	14.15	0.99 ± 0.14	13.88	0.42 ± 0.03	6.16	1.19 ± 0.02	1.98	0.62 ± 0.13	21.16	0.80 ± 0.02	2.67
ACC	4.53 ± 1.05	23.16	0.78 ± 0.11	14.81	0.17 ± 0.02	9.69	1.27 ± 0.07	5.89	1.02 ± 0.17	16.92	1.08 ± 0.08	7.06
Amyg	3.89 ± 1.31	33.66	0.62 ± 0.20	32.66	0.21 ± 0.02	9.49	1.07 ± 0.04	3.79	0.80 ± 0.15	19.33	0.88 ± 0.04	4.25
Caud	3.97 ± 1.19	29.94	0.79 ± 0.10	12.34	0.19 ± 0.02	9.83	1.12 ± 0.09	8.13	0.87 ± 0.16	17.88	0.95 ± 0.07	7.78
CGC	6.19 ± 1.11	17.92	0.93 ± 0.13	13.54	0.42 ± 0.05	11.06	1.25 ± 0.06	4.66	0.62 ± 0.12	19.62	0.82 ± 0.02	2.51
Hippo	3.98 ± 1.07	26.94	0.79 ± 0.15	18.69	0.18 ± 0.01	8.15	1.26 ± 0.07	5.42	0.98 ± 0.18	18.65	1.06 ± 0.06	5.61
INS	3.16 ± 0.64	20.32	0.96 ± 0.12	12.81	0.17 ± 0.02	8.91	1.47 ± 0.14	9.25	1.17 ± 0.21	18.26	1.23 ± 0.12	9.82
NucAcc	4.71 ± 1.43	30.46	0.59 ± 0.10	17.01	0.23 ± 0.03	12.84	1.03 ± 0.11	10.94	0.76 ± 0.15	19.46	0.83 ± 0.07	8.11
PCC	4.80 ± 1.10	22.83	0.87 ± 0.12	13.41	0.21 ± 0.02	10.37	1.18 ± 0.05	3.90	0.86 ± 0.16	18.80	0.95 ± 0.05	5.01
Put	7.28 ± 1.15	15.80	0.87 ± 0.11	13.11	0.24 ± 0.02	9.21	0.94 ± 0.03	2.82	0.66 ± 0.12	17.48	0.74 ± 0.02	2.35
Thal	7.82 ± 0.96	12.33	1.03 ± 0.15	14.11	0.33 ± 0.02	3.55	1.29 ± 0.06	5.01	0.81 ± 0.15	18.74	0.96 ± 0.06	5.66

Note: Raw summary metrics (MWF, T1w/T2w ratio, FA, AD, RD, MD) – broken down for every participant and every ROI – have been made freely available in a supplementary spreadsheet accompanying this article.

### Quantitative values in WM vs. subcortical GM structures

**Figure 3:**
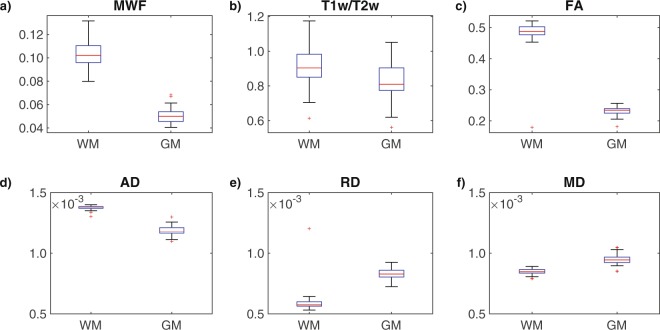
The box-and-whisker plots illustrate the comparison between WM and GM for the MRI measures (**a**) MWF, (**b**) T1w/T2w ratio, (**c**) FA, (**d**) AD, (**e**) RD, and (**f**) MD.

Figure 3 shows box and whisker plots for each MRI metric, broken down by tissue type (i.e., the mean values from the 15 WM structures and 10 GM structures). As expected, the paired-sample, one-tailed t-tests revealed that WM structures had significantly higher MWF (t = 40.07), T1w/T2w (t = 13.07), FA (t = 27.97) and AD (t = 24.91) values compared to subcortical GM structures, and significantly lower RD (t = −30.89) and MD (t = −18.46), all with p < 10^−10^.

### Correlations between quantitative MRI measures

Pearson (linear) correlations between every combination of MRI measures are presented for each brain structure in Table 2, and the corresponding Spearman (rank) correlations are presented in Table 3. Based on a comparison of these tables, the Pearson and Spearman correlations were in close agreement – where 34/375 (9.1%) Spearman correlations were found to be uniquely significant and only 2/375 (0.5%) Pearson correlations were found to be uniquely significant. To summarize, both Pearson and Spearman correlations were found to be significant between MD vs. AD for the majority of the 25 brain structures investigated, and the differences between Pearson and Spearman analyses were primarily due to uniquely significant rank (Spearman) correlations between RD vs. AD in 11/25 ROIs, between MD vs. RD in 19/25 ROIs and between RD vs. FA in 3/25 ROIs (Tables 2 and 3). Neither Pearson nor Spearman correlations between different MRI measures were found to be statistically significant in more than 1/25 brain structures after Bonferroni correcting for the number of between-metric comparisons (p < 0.003).

**Table 2:** Pearson correlations between different MRI measures (MWF, T1w/T2w, FA, AD, RD and MD) for each structure.

Pearson	T1w/T2w vs. MWF	FA vs. MWF	AD vs. MWF	RD vs. MWF	MD vs. MWF	FA vs. T1w/T2w	AD vs. T1w/T2w	RD vs. T1w/T2w	MD vs. T1w/T2w	AD vs. FA	RD vs. FA	MD vs. FA	RD vs. AD	MD vs. AD	MD vs. RD
ACR	0.06 (0.746)	−0.01 (0.975)	0.10 (0.589)	0.12 (0.524)	0.05 (0.789)	−0.05 (0.79)	0.12 (0.515)	0.12 (0.516)	0.11 (0.545)	−0.02 (0.907)	−0.07 (0.725)	−0.21 (0.259)	0.23 (0.214)	0.40 (0.027)	**0.54** (**0.002)**
ALIC	0.19 (0.307)	−0.13 (0.476)	−0.13 (0.484)	−0.07 (0.724)	−0.27 (0.139)	0.16 (0.388)	0.09 (0.624)	0.11 (0.565)	−0.15 (0.44)	0.48 (0.006)	−0.16 (0.402)	−0.36 (0.048)	−0.18 (0.341)	0.16 (0.388)	0.19 (0.297)
BCC	−0.09 (0.633)	−0.01 (0.953)	0.06 (0.742)	−0.05 (0.811)	−0.09 (0.65)	0.09 (0.638)	−0.26 (0.163)	0.08 (0.664)	−0.16 (0.379)	−0.02 (0.904)	−0.08 (0.661)	−0.17 (0.351)	0.20 (0.284)	**0.77** (**<10**^**−4**^**)**	0.40 (0.025)
CP	0.22 (0.232)	−0.13 (0.477)	−0.10 (0.592)	−0.21 (0.25)	−0.27 (0.15)	0.08 (0.69)	−0.16 (0.405)	0.11 (0.548)	−0.07 (0.702)	−0.16 (0.399)	−0.19 (0.317)	−0.34 (0.062)	0.38 (0.037)	**0.84** (**<10**^**−4**^**)**	**0.52** (**0.003)**
EC	−0.09 (0.647)	−0.02 (0.898)	−0.08 (0.673)	0.09 (0.648)	−0.04 (0.847)	0.21 (0.25)	−0.16 (0.406)	0.05 (0.778)	−0.18 (0.333)	−0.19 (0.319)	−0.12 (0.517)	−0.31 (0.093)	0.33 (0.069)	**0.77** (**<10**^**−4**^**)**	0.49 (0.006)
GCC	0.02 (0.904)	−0.13 (0.479)	0.09 (0.632)	0.11 (0.554)	0.06 (0.752)	0.01 (0.943)	−0.19 (0.3)	0.28 (0.123)	0.07 (0.728)	0.00 (0.988)	−0.07 (0.719)	−0.23 (0.218)	−0.19 (0.313)	0.49 (0.005)	0.22 (0.239)
IFO	0.06 (0.734)	−0.11 (0.571)	−0.13 (0.481)	0.15 (0.437)	0.07 (0.71)	0.00 (0.997)	−0.22 (0.234)	0.18 (0.331)	−0.02 (0.927)	0.44 (0.013)	−0.15 (0.413)	−0.17 (0.362)	0.06 (0.77)	0.27 (0.146)	0.51 (0.004)
PCR	−0.15 (0.413)	−0.17 (0.375)	0.02 (0.906)	−0.09 (0.616)	−0.08 (0.666)	0.00 (0.997)	0.05 (0.787)	0.10 (0.595)	0.11 (0.554)	−0.01 (0.975)	−0.09 (0.63)	0.06 (0.745)	0.10 (0.582)	**0.76** (**<10**^**−4**^**)**	0.54 (0.011)
PLIC	0.30 (0.106)	−0.04 (0.849)	0.20 (0.272)	−0.07 (0.722)	−0.11 (0.559)	0.12 (0.518)	−0.10 (0.595)	0.15 (0.415)	0.00 (0.998)	0.12 (0.514)	0.01 (0.95)	0.03 (0.867)	−0.07 (0.723)	**0.64** (**<10**^**−4**^**)**	0.19 (0.319)
PTR	0.03 (0.858)	0.12 (0.511)	−0.46 (0.009)	−0.37 (0.043)	−**0.62** (**<10**^**−4**^**)**	0.17 (0.348)	−0.05 (0.793)	0.06 (0.741)	−0.08 (0.657)	−0.19 (0.307)	−0.12 (0.528)	−0.23 (0.223)	0.29 (0.108)	**0.66** (**<10**^**−4**^**)**	**0.52** (**0.003)**
RLIC	0.14 (0.457)	−0.13 (0.501)	−0.20 (0.278)	−0.02 (0.916)	−0.49 (0.005)	0.02 (0.934)	−0.07 (0.713)	0.20 (0.292)	0.10 (0.575)	0.18 (0.346)	−0.01 (0.969)	−0.05 (0.775)	0.19 (0.312)	**0.67** (**<10**^**−4**^**)**	0.35 (0.057)
SCC	0.09 (0.638)	−0.12 (0.527)	0.01 (0.941)	−0.23 (0.207)	−0.10 (0.63)	0.23 (0.211)	−0.30 (0.102)	−0.11 (0.54)	−0.27 (0.15)	−0.33 (0.071)	−0.19 (0.303)	−0.37 (0.039)	0.35 (0.051)	**0.84** (**<10**^**−4**^**)**	**0.53** (**0.002)**
SCR	−0.12 (0.531)	0.04 (0.826)	−0.10 (0.603)	−0.05 (0.777)	−0.11 (0.544)	0.09 (0.619)	−0.17 (0.355)	0.05 (0.792)	−0.17 (0.349)	−0.05 (0.774)	0.00 (0.996)	−0.03 (0.86)	0.18 (0.347)	**0.74** (**<10**^**−4**^**)**	0.35 (0.056)
SFO	−0.37 (0.043)	−0.14 (0.457)	0.15 (0.43)	−0.02 (0.926)	0.10 (0.604)	−0.11 (0.561)	−0.21 (0.263)	0.08 (0.689)	0.02 (0.93)	0.31 (0.09)	−0.25 (0.184)	−0.40 (0.025)	0.22 (0.233)	**0.52** (**0.003)**	**0.57** (**0.001)**
SLF	−0.01 (0.973)	−0.13 (0.499)	0.00 (0.996)	−0.07 (0.709)	−0.18 (0.328)	0.06 (0.77)	0.08 (0.686)	0.14 (0.442)	0.17 (0.375)	0.09 (0.651)	−0.16 (0.406)	−0.17 (0.372)	0.17 (0.368)	0.37 (0.043)	**0.56** (**0.001)**
ACC	−0.10 (0.582)	0.20 (0.286)	0.18 (0.329)	0.08 (0.66)	0.19 (0.297)	−0.05 (0.802)	−0.22 (0.231)	0.06 (0.75)	−0.20 (0.271)	0.11 (0.549)	0.03 (0.894)	−0.03 (0.895)	0.28 (0.129)	**0.97** (**<10**^**−4**^**)**	0.31 (0.091)
Amyg	−0.27 (0.141)	−0.18 (0.321)	0.12 (0.52)	0.00 (0.984)	0.20 (0.265)	0.09 (0.627)	−0.10 (0.579)	0.23 (0.223)	−0.05 (0.794)	−0.19 (0.296)	−0.09 (0.62)	−0.42 (0.02)	0.37 (0.042)	**0.93** (**<10**^**−4**^**)**	0.38 (0.036)
Caud	−0.12 (0.527)	−0.12 (0.536)	−0.10 (0.591)	−0.22 (0.242)	−0.07 (0.705)	0.24 (0.203)	0.07 (0.844)	0.09 (0.649)	−0.05 (0.78)	0.27 (0.14)	0.20 (0.27)	0.17 (0.354)	0.40 (0.025)	**0.98** (**<10**^**−4**^**)**	0.34 (0.059)
CGC	−0.01 (0.98)	0.06 (0.752)	0.42 (0.019)	−0.01 (0.943)	−0.15 (0.424)	−0.04 (0.824)	−0.06 (0.748)	0.14 (0.457)	0.13 (0.502)	0.25 (0.182)	−0.04 (0.837)	0.02 (0.897)	−0.06 (0.743)	0.29 (0.109)	0.41 (0.023)
Hippo	−0.23 (0.218)	0.11 (0.55)	0.28 (0.123)	−0.03 (0.857)	0.26 (0.157)	−0.08 (0.689)	−0.13 (0.481)	0.14 (0.445)	−0.16 (0.381)	0.03 (0.872)	−0.11 (0.553)	−0.09 (0.625)	0.31 (0.091)	**0.98** (**<10**^**−4**^**)**	0.33 (0.075)
INS	−0.24 (0.199)	−0.12 (0.506)	0.31 (0.088)	0.29 (0.112)	0.26 (0.158)	0.10 (0.594)	−0.21 (0.261)	−0.02 (0.907)	−0.25 (0.169)	−0.21 (0.261)	−0.26 (0.167)	−0.28 (0.126)	0.44 (0.012)	**0.97** (**<10**^**−4**^**)**	0.49 (0.005)
NucAcc	0.06 (0.733)	−0.08 (0.688)	−0.06 (0.738)	−0.12 (0.505)	−0.12 (0.523)	−0.06 (0.751)	0.18 (0.321)	0.18 (0.328)	0.09 (0.619)	0.18 (0.322)	−0.10 (0.595)	0.05 (0.775)	0.42 (0.019)	**0.85** (**<10**^**−4**^**)**	0.41 (0.021)
PCC	−0.10 (0.598)	0.13 (0.482)	0.26 (0.166)	−0.17 (0.368)	0.14 (0.458)	−0.20 (0.287)	−0.07 (0.723)	0.05 (0.788)	−0.09 (0.63)	0.15 (0.421)	−0.18 (0.33)	−0.10 (0.605)	0.15 (0.411)	**0.84** (**<10**^**−4**^**)**	0.31 (0.09)
Put	0.12 (0.54)	0.07 (0.692)	−0.25 (0.181)	−0.11 (0.572)	−0.33 (0.069)	0.17 (0.375)	−0.15 (0.424)	0.12 (0.534)	−0.28 (0.125)	−0.01 (0.954)	−0.06 (0.769)	−0.37 (0.039)	0.05 (0.805)	**0.70** (**<10**^**−4**^**)**	0.12 (0.506)
Thal	0.26 (0.164)	−0.06 (0.731)	0.12 (0.535)	−0.09 (0.635)	0.11 (0.569)	0.23 (0.208)	0.02 (0.906)	0.12 (0.522)	−0.05 (0.803)	−0.24 (0.187)	0.07 (0.728)	−0.25 (0.17)	0.45 (0.012)	**0.98** (**<10**^**−4**^**)**	0.37 (0.041)

Note: Bold font indicates correlations that were statistically significant after correcting for multiple comparisons using a Bonferroni correction (p < 0.003).

**Table 3:** Spearman correlations between different MRI measures (MWF, T1w/T2w, FA, AD, RD and MD) for each structure.

Spearman	T1w/T2w vs. MWF	FA vs. MWF	AD vs. MWF	RD vs. MWF	MD vs. MWF	FA vs. T1w/T2w	AD vs. T1w/T2w	RD vs. T1w/T2w	MD vs. T1w/T2w	AD vs. FA	RD vs. FA	MD vs. FA	RD vs. AD	MD vs. AD	MD vs. RD
ACR	0.12 (0.527)	−0.12 (0.511)	0.17 (0.362)	0.04 (0.835)	0.10 (0.557)	−0.20 (0.27)	0.24 (0.201)	0.07 (0.705)	0.13 (0.481)	−0.07 (0.712)	−0.36 (0.047)	−0.39 (0.032)	−0.06 (0.764)	0.31 (0.087)	**0.90** (**<10**^−4^**)**
ALIC	0.25 (0.172)	−0.25 (0.178)	−0.15 (0.411)	−0.11 (0.575)	−0.26 (0.159)	0.09 (0.64)	0.09 (0.635)	−0.03 (0.878)	−0.06 (0.766)	**0.54** (**0.002)**	−**0.64** (**<10**^−4^**)**	−0.36 (0.047)	−0.51 (0.004)	0.14 (0.455)	**0.56** (**0.001)**
BCC	−0.14 (0.44)	0.15 (0.412)	0.04 (0.813)	−0.12 (0.524)	−0.09 (0.614)	−0.07 (0.697)	−0.24 (0.201)	−0.06 (0.744)	−0.15 (0.422)	0.06 (0.758)	−0.36 (0.049)	−0.23 (0.207)	0.38 (0.036)	**0.75** (**<10**^−4^**)**	**0.81** (**<10**^−4^**)**
CP	0.24 (0.204)	0.02 (0.901)	−0.04 (0.813)	−0.37 (0.041)	−0.32 (0.08)	−0.04 (0.841)	−0.24 (0.191)	−0.04 (0.833)	−0.22 (0.24)	0.08 (0.663)	−0.33 (0.068)	−0.22 (0.239)	**0.52** (**0.003)**	**0.73** (<**10**^**−4**^**)**	**0.90** (<**10**^**−4**^**)**
EC	−0.15 (0.417)	−0.17 (0.375)	0.00 (0.985)	0.19 (0.3)	0.16 (0.386)	0.25 (0.175)	−0.19 (0.319)	−0.15 (0.41)	−0.23 (0.212)	−0.12 (0.531)	−0.50 (0.005)	−0.41 (0.024)	0.35 (0.056)	**0.69** (**<10**^**−4**^**)**	**0.88** (**<10**^**−4**^**)**
GCC	−0.06 (0.739)	−0.18 (0.336)	0.14 (0.471)	0.08 (0.661)	0.03 (0.855)	−0.12 (0.531)	−0.15 (0.433)	0.18 (0.334)	0.05 (0.784)	0.17 (0.364)	−0.21 (0.266)	−0.15 (0.437)	−0.25 (0.169)	0.47 (0.008)	**0.62** (**<10**^**−4**^**)**
IFO	0.05 (0.793)	−0.18 (0.336)	−0.14 (0.466)	0.21 (0.268)	0.08 (0.68)	−0.19 (0.31)	−0.15 (0.416)	0.24 (0.201)	0.07 (0.725)	0.44 (0.013)	−0.49 (0.005)	−0.21 (0.253)	−0.34 (0.063)	0.31 (0.095)	**0.71** (**<10**^**−4**^**)**
PCR	−0.14 (0.452)	−0.09 (0.638)	0.03 (0.86)	0.02 (0.917)	−0.07 (0.717)	−0.12 (0.511)	0.02 (0.931)	0.12 (0.539)	0.12 (0.51)	0.22 (0.24)	−0.18 (0.326)	0.06 (0.765)	0.22 (0.23)	**0.78** (**<10**^**−4**^**)**	**0.74** (**<10**^**−4**^**)**
PLIC	0.34 (0.066)	0.05 (0.793)	0.21 (0.252)	−0.29 (0.118)	−0.19 (0.313)	0.03 (0.887)	−0.11 (0.548)	0.14 (0.442)	−0.01 (0.952)	0.31 (0.094)	−0.39 (0.029)	−0.26 (0.16)	−0.16 (0.389)	0.47 (0.008)	**0.70** (**<10**^**−4**^**)**
PTR	0.05 (0.799)	0.16 (0.384)	−0.49 (0.005)	−0.43 (0.016)	−**0.53** (**0.002)**	0.00 (0.986)	0.03 (0.875)	−0.06 (0.745)	−0.08 (0.679)	−0.16 (0.385)	−0.43 (0.017)	−0.40 (0.026)	**0.54** (**0.002)**	**0.70** (**<10**^**−4**^**)**	**0.94** (**<10**^**−4**^**)**
RLIC	0.13 (0.504)	0.06 (0.761)	−0.22 (0.244)	−0.41 (0.022)	−0.48 (0.006)	−0.23 (0.219)	−0.04 (0.85)	0.19 (0.299)	0.10 (0.605)	0.32 (0.084)	−0.43 (0.015)	−0.12 (0.507)	0.05 (0.801)	**0.63** (**<10**^**−4**^**)**	**0.75** (**<10**^**−4**^**)**
SCC	−0.03 (0.86)	0.06 (0.74)	−0.05 (0.798)	−0.18 (0.343)	−0.12 (0.535)	0.03 (0.856)	−0.35 (0.056)	−0.23 (0.206)	−0.36 (0.048)	−0.31 (0.091)	−**0.61** (**<10**^−4^**)**	−**0.53** (**0.002)**	**0.61** (**<10**^**−4**^**)**	**0.83** (**<10**^**−4**^**)**	**0.91** (**<10**^**−4**^**)**
SCR	0.00 (0.99)	0.24 (0.193)	−0.08 (0.662)	−0.08 (0.674)	−0.08 (0.68)	0.03 (0.875)	−0.18 (0.345)	−0.10 (0.612)	−0.15 (0.431)	0.00 (0.981)	−0.30 (0.108)	−0.19 (0.312)	0.39 (0.032)	**0.69** (**<10**^**−4**^**)**	**0.91** (**<10**^**−4**^**)**
SFO	−0.26 (0.155)	−0.13 (0.49)	0.24 (0.192)	−0.02 (0.927)	0.19 (0.309)	−0.05 (0.793)	−0.23 (0.212)	−0.02 (0.92)	−0.04 (0.818)	0.31 (0.095)	−**0.59** (**<10**^**−4**^**)**	−0.44 (0.014)	0.12 (0.531)	0.49 (0.006)	**0.86** (**<10**^**−4**^**)**
SLF	0.03 (0.87)	−0.07 (0.715)	0.06 (0.755)	−0.12 (0.534)	−0.07 (0.693)	−0.13 (0.501)	0.10 (0.578)	0.27 (0.15)	0.19 (0.316)	0.09 (0.632)	−0.46 (0.01)	−0.27 (0.135)	0.07 (0.725)	0.43 (0.016)	**0.90** (**<10**^**−4**^**)**
ACC	0.09 (0.643)	0.29 (0.108)	0.12 (0.526)	0.20 (0.281)	0.14 (0.461)	−0.05 (0.806)	−0.19 (0.314)	−0.06 (0.766)	−0.20 (0.294)	0.04 (0.85)	−0.17 (0.379)	−0.12 (0.521)	**0.77** (**<10**^**−4**^**)**	**0.95** (**<10**^**−4**^**)**	**0.86** (**<10**^**−4**^**)**
Amyg	−0.19 (0.3)	−0.06 (0.737)	−0.05 (0.788)	−0.05 (0.794)	−0.03 (0.884)	0.08 (0.677)	−0.02 (0.902)	0.17 (0.376)	0.08 (0.682)	−0.11 (0.542)	−0.35 (0.052)	−0.26 (0.164)	**0.84** (**<10**^**−4**^**)**	**0.93** (**<10**^**−4**^**)**	**0.96** (**<10**^**−4**^**)**
Caud	−0.10 (0.612)	−0.17 (0.336)	0.04 (0.836)	−0.06 (0.741)	0.04 (0.813)	0.09 (0.643)	−0.01 (0.949)	−0.07 (0.729)	−0.13 (0.484)	0.29 (0.116)	0.26 (0.152)	0.20 (0.285)	**0.90** (**<10**^**−4**^**)**	**0.97** (**<10**^**−4**^**)**	**0.90** (**<10**^**−4**^**)**
CGC	−0.05 (0.801)	0.18 (0.339)	0.38 (0.038)	0.33 (0.072)	−0.12 (0.507)	−0.19 (0.306)	−0.07 (0.722)	0.26 (0.163)	0.13 (0.48)	0.45 (0.01)	−0.41 (0.023)	−0.06 (0.747)	−0.46 (0.009)	0.25 (0.183)	**0.67** (**<10**^**−4**^**)**
Hippo	−0.21 (0.252)	0.36 (0.047)	0.05 (0.798)	−0.03 (0.856)	0.04 (0.834)	−0.01 (0.947)	−0.13 (0.503)	−0.10 (0.592)	−0.16 (0.403)	0.00 (0.997)	−0.18 (0.323)	−0.10 (0.606)	**0.94** (**<10**^**−4**^**)**	**0.98** (**<10**^**−4**^**)**	**0.95** (**<10**^**−4**^**)**
INS	−0.13 (0.504)	0.04 (0.839)	0.23 (0.221)	0.27 (0.139)	0.19 (0.313)	0.00 (0.988)	−0.22 (0.238)	−0.16 (0.395)	−0.26 (0.152)	−0.10 (0.597)	−0.23 (0.222)	−0.18 (0.323)	**0.84** (**<10**^**−4**^**)**	**0.95** (**<10**^**−4**^**)**	**0.90** (**<10**^**−4**^**)**
NucAcc	−0.02 (0.915)	−0.02 (0.905)	0.02 (0.937)	−0.11 (0.558)	−0.04 (0.817)	0.05 (0.789)	0.23 (0.221)	0.27 (0.143)	0.23 (0.214)	0.27 (0.14)	−0.08 (0.663)	0.12 (0.521)	**0.79** (**<10**^**−4**^**)**	**0.94** (**<10**^**−4**^**)**	**0.89** (**<10**^**−4**^**)**
PCC	−0.03 (0.887)	0.16 (0.388)	0.32 (0.081)	0.00 (0.988)	0.20 (0.28)	−0.18 (0.321)	−0.17 (0.374)	−0.05 (0.774)	−0.12 (0.531)	−0.02 (0.897)	−0.35 (0.056)	−0.25 (0.169)	**0.59** (**<10**^**−4**^**)**	**0.83** (**<10**^**−4**^**)**	**0.90** (**<10**^**−4**^**)**
Put	0.15 (0.418)	0.10 (0.582)	−0.19 (0.315)	−0.26 (0.152)	−0.23 (0.219)	0.20 (0.272)	−0.17 (0.354)	−0.15 (0.413)	−0.33 (0.067)	0.00 (0.986)	−0.45 (0.01)	−0.41 (0.023)	0.33 (0.069)	**0.69** (**<10**^**−4**^**)**	**0.83** (**<10**^**−4**^**)**
Thal	0.20 (0.275)	−0.04 (0.828)	0.22 (0.246)	0.13 (0.492)	0.22 (0.254)	0.22 (0.247)	−0.03 (0.868)	−0.06 (0.759)	−0.14 (0.453)	−0.10 (0.582)	−0.13 (0.501)	−0.20 (0.291)	**0.96** (**<10**^**−4**^**)**	**0.98** (**<10**^**−4**^**)**	**0.94** (**<10**^**−4**^**)**

Note: Bold font indicates correlations that were statistically significant after correcting for multiple comparisons using a Bonferroni correction (p < 0.003).

**Figure 4:**
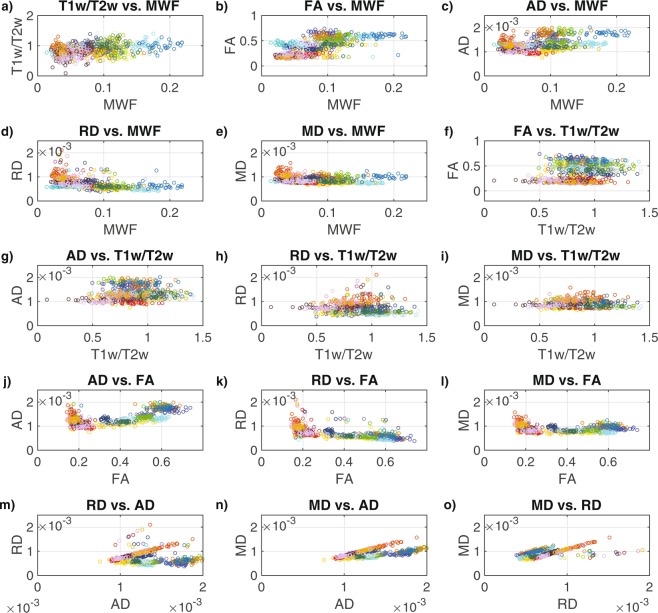
Scatter plots between the MRI measures across all 25 bilateral ROIs: (**a**) T1w/T2w vs. MWF, (**b**) FA vs. MWF, (**c**) AD vs. MWF, (**d**) RD vs. MWF, (**e**) MD vs. MWF, (**f**) FA vs. T1w/T2w, (**g**) AD vs. Tw/T2w, (**h**) RD vs. Tw/T2w, (**i**) MD vs. Tw/T2w, (**j**) AD vs. FA, (**k**) RD vs. FA, (**l**) MD vs. FA, (**m**) RD vs. AD, (**n**) MD vs. AD and (**o**) MD vs. RD. Each structure is indicated by a different color in order to match the ROIs shown in Fig. 1, and the values for each participant are represented by individual dots.

However, both Pearson and Spearman correlations of the data combined across all 31 participants and all 25 structures (Fig. 4, Table 4) revealed positive correlations for T1w/T2w vs. MWF (r = 0.33, ρ = 0.31), FA vs. MWF (r = 0.73, ρ = 0.75), FA vs. T1w/T2w (r = 0.25, ρ = 0.22), MD vs. AD (r = 0.75, ρ = 0.84), AD vs. MWF (r = 0.43, ρ = 0.36), AD vs. T1w/T2w (r = 0.14, ρ = 0.20), AD vs. FA (r = 0.65, ρ = 0.59), MD vs. AD (r = 0.57, ρ = 0.58), MD vs. RD (r = 0.64, ρ = 0.61), and negative correlations for RD vs. MWF (r = −0.49, ρ = −0.62), RD vs. FA (r = −0.62, ρ = −0.75), MD vs. MWF (r = −0.22, ρ = −0.29) and MD vs. FA (r = −0.22, ρ = −0.18) – all of which were statistically significant after Bonferroni correcting for the number of between-metric comparisons (p < 0.003). Although, the Spearman correlations for T1w/T2w vs. RD (ρ = −0.14) and AD vs. RD (ρ = −0.17) were statistically significant, the corresponding Pearson correlations were not; and the relationship for MD vs. T1w/T2w was not significant based on either Pearson or Spearman correlations.

**Table 4:** Pearson (lower triangle) and Spearman (upper triangle) correlation coefficients with p-values (indicated in parentheses) between different MRI measures across all 25 structures.

All	MWF	T1w/T2w	FA	AD	RD	MD
MWF		**0.311 (<10** ^**−6**^ **)**	**0.748 (<10** ^**−6**^ **)**	**0.359 (<10** ^**−6**^ **)**	**−0.623 (<10** ^**−6**^ **)**	**−0.285 (<10** ^**−6**^ **)**
T1w/T2w	**0.326 (<10** ^**−6**^ **)**		**0.216 (<10** ^**−6**^ **)**	**0.199 (<10** ^**−6**^ **)**	**−0.140 (<10** ^**−6**^ **)**	−0.058 (0.106)
FA	**0.727 (<10** ^**−6**^ **)**	**0.245 (<10** ^**−6**^ **)**		**0.589 (<10** ^**−6**^ **)**	**−0.753 (<10** ^**−6**^ **)**	**−0.184 (<10** ^**−6**^ **)**
AD	**0.428 (<10** ^**−6**^ **)**	**0.144 (<10** ^**−6**^ **)**	**0.654 (<10** ^**−6**^ **)**		**−0.170 (<10** ^**−6**^ **)**	**0.576 (<10** ^**−6**^ **)**
RD	**−0.486 (<10** ^**−6**^ **)**	−0.070 0.050	**−0.618 (<10** ^**−6**^ **)**	−0.052 (0.150)		**0.606 (<10** ^**−6**^ **)**
MD	**−0.222 (<10** ^**−6**^ **)**	−0.038 (0.285)	**−0.224 (<10** ^**−6**^ **)**	**0.569 (<10** ^**−6**^ **)**	**0.637 (<10** ^**−6**^ **)**	

Corresponding data plots are shown in Fig. 4. Note: Bold font indicates correlations that were statistically significant after correcting for multiple comparisons using a Bonferroni correction (p < 0.003).

**Figure 5:**
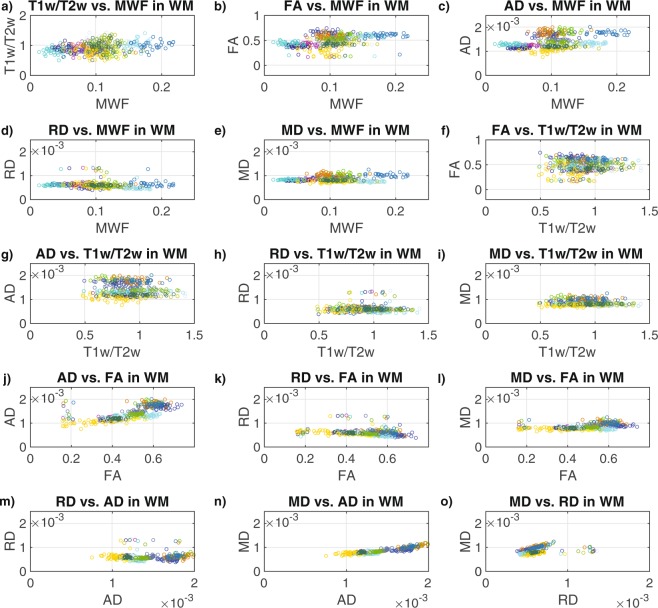
Scatter plots between the MRI measures across the 15 bilateral white matter ROIs: (**a**) T1w/T2w vs. MWF in WM (**b**) FA vs. MWF in WM, (**c**) AD vs. MWF in WM, (**d**) RD vs. MWF in WM, (**e**) MD vs. MWF in WM, (**f**) FA vs. T1w/T2w in WM, (**g**) AD vs. Tw/T2w in WM, (**h**) RD vs. Tw/T2w in WM, (**i**) MD vs. Tw/T2w in WM, (**j**) AD vs. FA in WM, (**k**) RD vs. FA in WM, (**l**) MD vs. FA in WM, (**m**) RD vs. AD in WM, (**n**) MD vs. AD in WM, and (**o**) MD vs. RD in WM. Each structure is indicated by a different color in order to match the ROIs shown in Fig. 1, and the values for each participant are represented by individual dots.

Similarly, both Pearson and Spearman correlations of the data combined across all 31 participants and the 15 WM structures (Fig. 5, Table 5) revealed significantly positive correlations for T1w/T2w vs. MWF (r = 0.25, ρ = 0.22), FA vs. MWF (r = 0.39, ρ = 0.43), AD vs. MWF (r = 0.36, ρ = 0.33), AD vs. FA (r = 0.71, ρ = 0.80), MD vs. FA (r = 0.45, ρ = 0.48), MD vs. AD (r = 0.89, ρ = 0.81) and MD vs. RD (r = 0.28, ρ = 0.39), and a negative correlation for RD vs. FA (r = −0.21, ρ = −0.39). Although the Spearman correlation for RD vs. MWF (r = −0.26) was statistically significant, the corresponding Pearson correlation was not; and although the Pearson correlation for MD vs. MWF (r = 0.21) was statistically significant, the corresponding Spearman correlation was not. Relationships among WM structures for FA vs. T1w/T2w, AD vs. T1w/T2w, RD vs. T1w/T2w, MD vs. T1w/T2w and RD vs. AD were not significant based on either Pearson or Spearman correlations.

**Table 5:** Pearson (lower triangle) and Spearman (upper triangle) correlation coefficients with p−values (indicated in parentheses) between different MRI measures across all 15 white matter structures.

WM	MWF	T1w/T2w	FA	AD	RD	MD
MWF		**0.223 (<10** ^**−6**^ **)**	**0.433 (<10** ^**−6**^ **)**	**0.331 (<10** ^**−6**^ **)**	**−0.263 (<10** ^**−6**^ **)**	0.052 (0.263)
T1w/T2w	**0.247 (<10** ^**−6**^ **)**		−0.005 (0.922)	−0.021 (0.659)	0.041 (0.375)	−0.029 (0.526)
FA	**0.385 (<10** ^**−6**^ **)**	0.040 (0.387)		**0.800 (<10** ^**−6**^ **)**	**−0.389 (<10** ^**−6**^ **)**	**0.478 (<10** ^**−6**^ **)**
AD	**0.358 (<10** ^**−6**^ **)**	−0.059 (0.204)	**0.712 (<10** ^**−6**^ **)**		−0.075 (0.104)	**0.814 (<10** ^**−6**^ **)**
RD	−0.123 (0.008)	0.068 (0.141)	**−0.208 (<10** ^**−6**^ **)**	0.026 (0.582)		**0.387 (<10** ^**−6**^ **)**
MD	**0.210 (<10** ^**−6**^ **)**	−0.044 (0.349)	**0.448 (<10** ^**−6**^ **)**	**0.892 (<10** ^**−6**^ **)**	**0.279 (<10** ^**−6**^ **)**	

Corresponding data plots are shown in Fig. 5. Note: Bold font indicates correlations that were statistically significant after correcting for multiple comparisons using a Bonferroni correction (p < 0.003).

**Figure 6:**
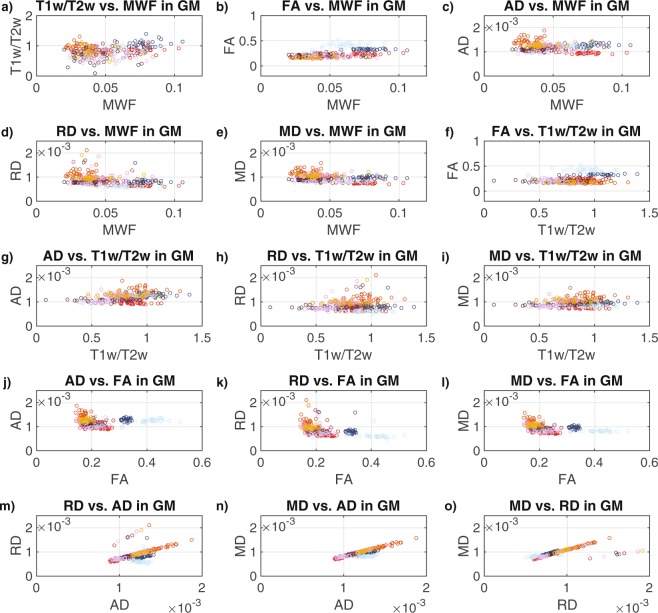
Scatter plots between the MRI measures across the 10 bilateral grey matter ROIs: (**a**) T1w/T2w vs. MWF in GM (**b**) FA vs. MWF in GM, (**c**) AD vs. MWF in GM, (**d**) RD vs. MWF in GM, (**e**) MD vs. MWF in GM, (**f**) FA vs. T1w/T2w in GM, (**g**) AD vs. Tw/T2w in GM, (**h**) RD vs. Tw/T2w in GM, (**i**) MD vs. Tw/T2w in GM, (**j**) AD vs. FA in GM, (**k**) RD vs. FA in GM, (**l**) MD vs. FA in GM, (**m**) RD vs. AD in GM, (**n**) MD vs. AD in GM, and (**o**) MD vs. RD in GM. Each structure is indicated by a different color in order to match the ROIs shown in Fig. 1, and the values for each participant are represented by individual dots.

Finally, both Pearson and Spearman correlations of the data combined across all 31 participants and the 10 GM structures (Fig. 6, Table 6) revealed significantly positive correlations for T1w/T2w vs. MWF (r = 0.25, ρ = 0.20), FA vs. MWF (r = 0.53, ρ = 0.55), FA vs. T1w/T2w (r = 0.29, ρ = 0.21), AD vs. T1w/T2w (r = 0.34, ρ = 0.35), RD vs. AD (r = 0.55, ρ = −0.60), MD vs. AD (r = 0.86, ρ = 0.80) and MD vs. RD (r = 0.73, ρ = 0.90), and negative correlations for RD vs. MWF (r = −0.40, ρ = −0.48), RD vs. FA (r = −0.48, ρ = −0.70), MD vs. MWF (r = −0.40, ρ = −0.41), MD vs. FA (r = −0.45, ρ = −0.62). Although the Spearman correlation for AD vs. FA (ρ = −0.19) was statistically significant, the corresponding Pearson correlation was not; and relationships among GM structures for AD vs. MWF, RD vs. T1w/T2w and MD vs. T1w/T2w were not significant based on either Pearson or Spearman correlations.

**Table 6:** Pearson (lower triangle) and Spearman (upper triangle) correlation coefficients with p-values (indicated in parentheses) between different MRI measures across all 10 grey matter structures. Corresponding data plots are shown in Fig. 6.

GM	MWF	T1w/T2w	FA	AD	RD	MD
MWF		**0.203 (<10** ^**−6**^ **)**	**0.554 (<10** ^**−6**^ **)**	−0.117 (0.040)	**−0.479 (<10** ^**−6**^ **)**	**−0.407 (<10** ^**−6**^ **)**
T1w/T2w	**0.245 (<10** ^**−6**^ **)**		**0.210 (<10** ^**−6**^ **)**	**0.353 (<10** ^**−6**^ **)**	0.037 (0.520)	0.128 (0.024)
FA	**0.528 (<10** ^**−6**^ **)**	**0.287 (<10** ^**−6**^ **)**		**−0.192 (0.001)**	**−0.696 (<10** ^**−6**^ **)**	**−0.616 (<10** ^**−6**^ **)**
AD	−0.165 (0.004)	**0.341 (<10** ^**−6**^ **)**	0.006 (0.919)		**0.603 (<10** ^**−6**^ **)**	**0.802 (<10** ^**−6**^ **)**
RD	**−0.397 (<10** ^**−6**^ **)**	0.108 (0.057)	**−0.482 (<10** ^**−6**^ **)**	**0.547 (<10** ^**−6**^ **)**		**0.903 (<10** ^**−6**^ **)**
MD	**−0.401 (<10** ^**−6**^ **)**	0.152 (0.007)	**−0.451 (<10** ^**−6**^ **)**	**0.863 (<10** ^**−6**^ **)**	**0.730 (<10** ^**−6**^ **)**	

Note: Bold font indicates correlations that were statistically significant after correcting for multiple comparisons using a Bonferroni correction (p < 0.003).

### Comparison of z-scored MRI metrics between brain regions

**Figure 7:**
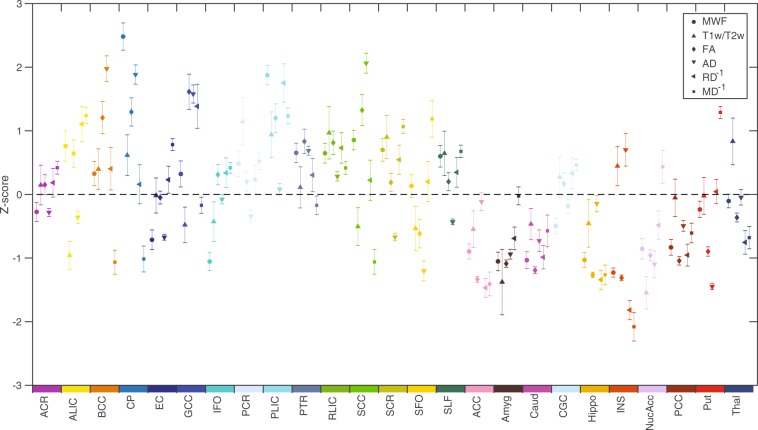
Standardized z-scores (mean ± 99% confidence interval) computed across the brain are shown for each measure (MWF, T1w/T2w, FA, AD, RD^−1^ and MD^−1^) in each of the 25 ROIs. Each structure is indicated by a different color in order to match the ROIs shown in Fig. 1, and values for RD and MD were inverted (RD^−1^ and MD^−1^) prior to z-scoring, in order to produce analogous contrasts (i.e., positive z-scores reflecting greater microstructural integrity and negative z-scores reflecting lower microstructural integrity) across measures.

Figure 7 presents the means and 99% confidence intervals (CI) for all six MRI measures (broken down by ROI) as standardized z-scores across all 25 brain structures. Although, some of the structures (e.g., ACR, PTR, RLIC, Caud, GCG, PCC) showed reasonably good correspondence between metrics (as indicated by differences in mean z-scores < 1 and many overlapping 99% CIs), several other regions (e.g., BCC, CP, GCC, SCC, SFO, INS, Put) exhibit poor correspondence between metrics (as indicated by differences in mean z-scores > 2 and relatively few overlapping 99% CIs). Furthermore, no consistent relationships between the six metrics were observed. For example, the body of the corpus callosum (BCC) appeared to have an average MWF (z-score ≈ 0.25), high FA (z-score ≈ 1.25) and low MD^−1^ (z-score ≈ −1.00) compared to other structures; the cerebral peduncles (CP; displayed immediately to the right of the BCC in Fig. 7) appeared to have an extremely high MWF (z-score ≈ 2.50), high FA (z-score ≈ 1.25) and low MD^−1^ (z-score ≈ −1.00); and the external capsule (EC; displayed immediately to the right of the CP in Fig. 7) appeared to have a slightly lower than average MWF (z-score ≈ −0.75), average FA (z-score ≈ 0.00) and slightly higher than average MD^−1^ (z-score ≈ 0.75).

These observations (from Fig. 7), were further underscored by the repeated measures ANOVAs, to formally compare the mean z-scores (from all 6 MRI metrics) within each region. The resulting F-statistics (Table 7), revealed that at least one of the MRI measures was significantly different from the others in all 25 ROIs, even after Bonferroni correction – suggesting that the different MRI measures have different sensitivities and/or specificities to the underlying tissue properties in different brain regions.

**Table 7:** A Repeated measure ANOVA from z-score across six different MRI measures (i.e., MWF, T1w/T2w, FA, AD, RD, and MD). Corresponding data plots are shown in Fig. 7.

Structures	F	p
ACR	**14.98**	**<10** ^**−6**^
ALIC	**136.87**	**<10** ^**−6**^
BCC	**107.55**	**<10** ^**−6**^
CP	**189.27**	**<10** ^**−6**^
EC	**78.76**	**<10** ^**−6**^
GCC	**97.76**	**<10** ^**−6**^
IFO	**63.11**	**<10** ^**−6**^
PCR	**33.32**	**<10** ^**−6**^
PLIC	**54.14**	**<10** ^**−6**^
PTR	**25.89**	**<10** ^**−6**^
RLIC	**8.58**	**<10** ^**−6**^
SCC	**176.35**	**<10** ^**−6**^
SCR	**68.32**	**<10** ^**−6**^
SFO	**70.09**	**<10** ^**−6**^
SLF	**30.58**	**<10** ^**−6**^
ACC	**63.58**	**<10** ^**−6**^
Amyg	**24.11**	**<10** ^**−6**^
Caud	**14.93**	**<10** ^**−6**^
CGC	**24.01**	**<10** ^**−6**^
Hippo	**43.32**	**<10** ^**−6**^
INS	**207.81**	**<10** ^**−6**^
NucAcc	**66.75**	**<10** ^**−6**^
PCC	**31.81**	**<10** ^**−6**^
Put	**240.61**	**<10** ^**−6**^
Thal	**57.36**	**<10** ^**−6**^

Note: Bold font indicates correlations that were statistically significant after correcting for multiple comparisons using a Bonferroni correction (p < 0.002).

## Discussion

In this cross-sectional study, we have measured and characterized relationships between six quantitative brain MRI measures, including multi-component T2-based MWF, T1w/T2w ratio, and four different DTI measures (i.e., FA, AD, RD and MD) in a cohort of 31 neurologically healthy participants. Our results confirmed that the values of all six methods showed significant differences between WM and subcortical GM structures, where MWF, T1w/T2w, FA and AD values were significantly higher in WM regions and both RD and MD values were significantly higher in subcortical GM regions (Fig. 3). Our work also indicated that these six measures were generally correlated with each other (to varying degrees) across all participants and the 15 WM and/or 10 subcortical GM structures examined in the current study. However, correlations were generally found to be less statistically significant within individual ROIs (except for the three DTI diffusivity metrics, which were significantly inter-related based on Spearman correlations); and z-scoring the values within each metric did not reveal consistent relationships (across brain regions) between many of the metrics. Taken together, this suggests that, while all six measures are indicators of brain microstructure, different metrics are sensitive to distinct combinations of tissue characteristics and/or local artifacts.

Consistent with previous work^48^, our results indicate that FA (which measures the directional homogeneity of water diffusion) had the strongest and most significant correlations with MWF (which is thought to be the most myelin-specific measure) after pooling data across all 25 structures (Fig. 4, Table 4), the 15 WM structures (Fig. 5, Table 5) and the 10 GM structures (Fig. 6, Table 6) – with T1w/T2w ratio also showing significant positive correlations with MWF after pooling data across all 25 structures, the 15 WM structures and the 10 GM structures. Specifically, FA and MWF had 53% shared variance among all 25 brain structures (r^2^ = 0.53), 15% shared variance among WM structures (r^2^ = 0.15) and 28% shared variance among GM structures (r^2^ = 0.28), implying that a reasonably large proportion of FA values could conceivably be related to myelin (noting the inherent limitations of correlation vs. causation). On the other hand, T1w/T2w ratios only shared 11% variance with MWF among all brain structures (r^2^ = 0.11), 6% among WM structures (r^2^ = 0.06), and 6% among GM structures (r^2^ = 0.06), suggesting that T1w/T2w ratio measurements are likely dominated by factors other than myelination – a finding that is consistent with other recent work^38,39^. Nevertheless, although the shared variance was small between T1w/T2w ratio and MWF, the statistical significance of these correlations is due to the large number of data points included in the correlations (e.g., 31 participants × 25 ROIs = 775 data points), suggesting that myelination contributes to a small but statistically significant portion of T1w/T2w ratio measurements.

As expected, pooling data across all 25 structures yielded a significant positive correlation for AD vs. MWF and significant negative correlations for RD vs. MWF and MD vs. MWF (based on both Pearson and Spearman correlations); and pooling data across the 15 WM ROIs yielded a significantly positive Spearman correlation for AD vs. MWF and a significantly negative Spearman correlation for RD vs. MWF. However, pooling data across the 10 subcortical GM ROIs yielded significantly negative correlations for RD vs. MWF and MD vs. MWF (based on both Pearson and Spearman correlations), with no significant relationships identified between AD and MWF.

Overall, the relationship between FA vs. MWF (positive correlation) was found to have the largest effect size across all 25 brain structures, and the next largest effect sizes were observed for AD vs. FA (positive correlation), RD vs. FA (negative correlation), MD vs. AD (positive correlation) and MD vs. RD (positive correlation) – all of which had more than 25% shared variance (r^2^ > 0.25). Therefore, combined with the many significant within-ROI Spearman correlations between AD, RD and MD (Table 3), it appears – perhaps not surprisingly – as though the four diffusion metrics (particularly the three diffusivity measures) reflect largely similar, albeit not identical, characteristics of the underlying tissues.

The fact that relatively few correlations were found within individual structures likely has two primary causes. The first is that our study sample was comprised of neurologically-healthy control participants, who presumably have relatively consistent tissue microstructure within any given brain region (e.g., compared to patient populations). Thus, if there is a small range of values along either (or both) dimension(s), then any true correlations are more likely to be obscured by variance owing to small amounts of measurement error (e.g., due to signal noise limitations, etc.). Moreover, from a statistical standpoint, the measurements within each region have much lower power because the sample size (i.e., number of data points = number of participants = 31) is much smaller than correlations across several different brain structures (e.g., number of data points = number of participants × number of ROIs = 775 across all 25 brain regions) [see Study Limitations section below for more details].

Nonetheless, as expected, WM structures (on average) had significantly higher MWF, T1w/T2w ratio, FA and AD – and lower RD and MD values – compared to GM structures (Fig. 3). However, by comparing the z-scored values of all six MRI measures across different structures (Fig. 7), there do not appear to be any systematic relationships between the various metrics (i.e., any particular metric being high or low did not consistently predict whether any other metric is high or low across structures), indicating their sensitivities to different underlying factors. For example, the observation of high MWF (z-score ≈ 2.50) and high FA (z-score ≈ 1.25) values in the cerebral peduncles (CP; collectively made up of the corticobulbar, corticopontine, and corticospinal fibers) is consistent with the fact that these structures are both highly myelinated and have well-organized fiber orientations. However, other structures known to have highly uniform fiber orientations (e.g., genu of the corpus callosum; GCC)^60^ exhibited relatively high FA (z-score ≈ 1.75) despite a moderate MWF (z-score ≈ 0.25), suggesting that the degree of fiber coherence contributes to FA more in this region than can be explained by myelin alone^41,61^. Interestingly, this same relationship held true for both the body and splenium of the corpus callosum (BCC and SCC) as well, while white matter structures that are known to have complex fiber geometries (e.g., superior corona radiata, superior fronto-occipital fasciculus and superior longitudinal fasciculus; SCR, SFO and SLF)^60^ – along with many of the subcortical GM ROIs – tended to have lower FA z-scores than MWF z-scores (as might have been expected).

Finally, although the current findings support previous reports that T1w/T2w ratio is not particularly specific to myelin concentration^38–40^, one of the other objectives of the study was to determine if T1w/T2w values might correlate better with other general measures of tissue microstructure, such as FA, AD, RD and/or MD values. Interestingly, however, the metric most highly correlated with T1w/T2w ratio across all 25 brain structures was MWF (r = 0.33), followed by FA (r = 0.25), and then AD (r = 0.14), corresponding to 11% shared variance with MWF, 6% shared variance with FA, and 2% shared variance with AD overall; and the shared variance between T1w/T2w vs. MWF and T1w/T2w vs. FA falls to 6% and 0% (respectively) across WM structures, and 6% and 8% (respectively) across GM structures. It is also noteworthy that all but 2 out of the 9 between-metric relationships that failed to reach statistical significance with either Pearson or Spearman correlations (Tables 4–6) – i.e., RD vs. AD in white matter (Table 5) and AD vs. MWF in gray matter (Table 6) – involved T1w/T2w ratios. Taken together, our results therefore suggest that: (1) T1w/T2w ratios are sensitive to unique aspects of tissue microstructure that are largely independent of either myelin-based or diffusion-based metrics, and (2) the differences between T1w/T2w ratios and the diffusion-based metrics are particularly striking among white matter regions (see Table 6).

### Study Limitations

As with any scientific experiment, the results of the current study must be interpreted within the context of known limitations.

First, although our sample size is comparable to similar previous studies^29,38–40,48–50^, it is worth noting that it is perhaps on the lower end for performing some of these types of comparisons. For example, one-tailed t-tests and/or correlations (since the general directional relationships between MWF, T1w/T2w ratio, FA, AD, RD and MD were already known) with sample sizes of n = 31 allow intermediate correlations with r ≥ 0.35 (or ρ ≥ 0.35) to be detected with 95% confidence and 80% power (i.e., alpha = 0.05 and beta = 0.20) for each comparison. Therefore, especially after correcting for multiple comparisons, statistical significance for within-ROI comparisons (Tables 2 and 3) could only be reached for large correlations with r ≥ 0.5 (or ρ ≥ 0.5). However, for correlations across brain structures (e.g., across 10 GM regions, 15 WM regions, or all 25 regions), it is worth pointing out that much smaller effect sizes become readily detectable (e.g., r ≥ 0.11 [uncorrected] or r ≥ 0.17 [corrected] for 31 participants × 10 regions = 310 data points) with the same statistical confidence and power.

Second, although we employed advanced image-processing steps – e.g., a well-validated LDDMM algorithm for high-dimensional nonlinear spatial normalization, bias correction and intensity calibration for the T1w/T2w ratio calculations, and stimulated echo correction for the MWF maps – we cannot rule out the possibility that small differences in coregistration, partial volume averaging or other effects due to imperfect preprocessing may have contributed some variability between imaging approaches. It is worth noting, however, that the ROI-based analyses used in the current study (i.e., averaging individual signals across entire brain structures) should mitigate these types of misalignment and/or partial volume effects compared to voxel-wise analysis approaches.

Third, MWF estimates in GM structures are likely to be less accurate than in WM structures due to inherently lower contrast-to-noise ratio (CNR), and MWF in the internal capsule may be slightly overestimated due to the use of the NNLS approach^62^ and an overlap between intra/extracellular water and myelin water signal in that region.

Fourth, a relatively short TR was used to acquire the GRASE images, thereby imparting a small degree of T1-weighting in otherwise T2w GRASE images used for MWF and T1w/T2w ratio calculations. However, other recent work in our lab has validated that T2w GRASE images can be used in order to calculate T1w/T2w ratios that are comparable to those obtained using FSE-based T2w images^38^.

Finally, given that the current experiment was purely empirical (i.e., measuring and comparing the different MRI values), we have avoided detailed discussion or speculation about the underlying mechanisms related to each imaging modality and what factors might be driving the observed differences between methods, beyond what is already well known. However, more thoroughly understanding the mechanisms underlying these signals is an important topic that has been explored by comparing various quantitative MRI approaches to *ex vivo* histological preparations – and there have been some very recent advances using optically cleared tissues that will likely open new avenues for better understanding what aspects of tissue microstructure these MRI signals are measuring at the cellular and sub-cellular levels^63^.

## Conclusions

Overall, all six of the evaluated quantitative MRI measures (MWF, T1w/T2w ratio, FA, AD, RD and MD) were found to be correlated with each other to varying degrees. The strongest correlation observed was between MWF and FA, which shared 53% variance across all 25 brain structures. However, the mean values (and z-scores across structures) differed between measures in several brain regions, and these differences can likely be attributed to unique sensitivities of the T1w/T2w ratios and diffusion-based measures to non-myelin factors, including: white matter fiber orientation (e.g., crossing fibers), proton density (e.g., tissue swelling), neural and glial density (e.g., axonal packing), iron/mineral accumulation, and/or local image artifacts. Importantly, the current study verifies previous work showing that calibrated T1w/T2w ratios differ from MWF estimates, and therefore should not be interpreted as myelin-specific measurements in subcortical brain structures; however, in revealing differences between T1w/T2w ratios and FA, AD and MD, it also appears that T1w/T2w ratios provide different/additional information about the underlying tissue characteristics compared to diffusion-based measurements. Given these differences, it stands to reason that a combination of methods may provide complimentary information. However, practical considerations such as available hardware and overall scan time are likely to dictate how many (and what types) of pulse sequences can be acquired in any given protocol; and although using multiple imaging modalities may shed additional light on brain development, aging and/or pathology, repeating statistical analyses using different quantitative MRI metrics can decrease statistical power (i.e., if corrections for type-I error due to multiple comparisons are required). Therefore, information about how the various metrics are related to each other can hopefully be used to inform decisions about what types of data to acquire and analyze in future multi-modal neuroimaging studies (e.g., to maximize sensitivity and/or specificity to certain brain pathologies while minimizing scan time and data redundancy).

## Supplementary information

Supplementary Dataset

## Data Availability

Unfortunately, the raw MRI data generated and analyzed in the current study cannot be made publicly available due to Research Ethics Board restrictions governing the storage and use of human neuroimaging data. However, anonymized images could potentially be made available upon special request from the corresponding author (pending the approval of the Research Ethics Board). Nonetheless, we have made our summary data – i.e., all raw MRI metrics (MWF, T1w/T2w ratio, FA, AD, RD, MD), broken down by participant and ROI – available as a supplementary spreadsheet accompanying this article.
